# RhoA inhibits the hypoxia-induced apoptosis and mitochondrial dysfunction in chondrocytes via positively regulating the CREB phosphorylation

**DOI:** 10.1042/BSR20160622

**Published:** 2017-04-20

**Authors:** Kai Zhang, Dianming Jiang

**Affiliations:** Department of Orthopaedics, First Affiliated Hospital of Chongqing Medical University, Chongqing 400016, China

**Keywords:** Apoptosis, Chondrocytes, CREB phosphorylation, Hypoxia, Mitochondrial dysfunction, RhoA

## Abstract

Chondrocytes that are embedded within the growth plate or the intervertebral disc are sensitive to environmental stresses, such as inflammation and hypoxia. However, little is known about the molecular signalling pathways underlining the hypoxia-induced mitochondrial dysfunction and apoptosis in chondrocytes. In the present study, we firstly examined the hypoxia-induced apoptosis, mitochondrial dysfunction and the activation of cyclic adenosine monophosphate (cAMP) response element-binding protein (CREB) signalling in human chondrocyte cell line, C28/I2 and then investigated the regulatory role of RhoA, a well-recognized apoptosis suppressor, in such process, with gain-of-function strategy. Our results indicated that hypoxia induced apoptosis and inhibited CREB phosphprylation in chondrocytes, meanwhile, the dysfunctional mitochondria with up-regulated mitochondrial superoxide and reactive oxygen species (ROS) levels, whereas with a reduced mitochondrial membrane potential (MMP) and Complex IV activity were observed in the hypoxia-treated C28/I2 cells. However, the overexpressed RhoA blocked the hypoxia-mediated reduction in CREB phosphprylation and inhibited the apoptosis induction, along with an ameliorated mitochondrial function in the hypoxia-treated C28/I2 cells. In conclusion, the present study confirmed the reduced CREB phosphorylation, along with the apoptosis induction and mitochondrial dysfunction in the hypoxia-treated chondrocyte cells. And the overexpression of RhoA ameliorated the hypoxia-induced mitochondrial dysfunction and apoptosis via blocking the hypoxia-mediated reduction in CREB phosphorylation.

## Introduction

Chondrocytes which are embedded within the growth plate or the intervertebral disc survive in an almost avascular and hypoxic milieu and are sensitive to environmental and multiple cellular stresses, such as inflammation, endoplasmic reticulum (ER) stress and hypoxia [1,2]. Hypertrophic chondrocytes die through the induction of programmed cell death (apoptosis) in the background of cervical spondylosis or the joint disorder containing osteoarthritis (OA) characterized by progressive breakdown of articular cartilage [3,4]. Reactive oxygen species (ROS) generated from the hypoxia/ischaemia trigger apoptotic cell death [5].

Mitochondria are the key targets of ROS, which have been suggested to regulate the processes involved in the mitochondrial dysfunction and in the promotion of apoptosis. The elevated ROS production causes mitochondrial damage, such as collapse of the mitochondrial membrane potential (MMP), complex IV inactivation, which opens up the mitochondrial permeability transition pores, leading to the release of cytochrome *c* (Cyt *c*) into the cytoplasm, to the cleavage of pro-apoptotic proteins [6,7]. Then the released Cyt *c* initiates the mitochondria-dependent caspase 3 (CASP 3) pathway that plays a pivotal role in the apoptotic cell death [6,7]. ROS also have indicated to cause structural and functional damage to mitochondria [10]. However, little is known about the molecular signalling pathways underlining the mitochondria-dependent caspase pathway.

Cyclic adenosine monophosphate (cAMP) response element-binding protein (CREB) is necessary for the cell proliferation and apoptosis [11] via regulating the expression of a repertoire of genes associated with cell survival, such as B-cell lymphoma 2 (Bcl-2), B-cell lymphoma-extra large (Bcl-xL) and c-*fos* [11], particularly in the condition of hypoxia [14]. And the inhibition of CREB-Ser^133^ phosphorylation results in the suppression of antiapoptotic genes [15,16]. Therefore, the CREB signalling might be implicated in the hypoxia-induced apoptosis in chondrocytes.

In the present study, we firstly examined the hypoxia-induced apoptosis and the activation of CREB signalling in human chondrocyte cell line, C28/I2, and then investigated the regulatory role of RhoA, a well-recognized apoptosis suppressor [17] in the hypoxia-promoted apoptosis in C28/I2 cells with gain-of-function strategy. Our results implied the involvement of CREB signalling and the protective role of RhoA in the hypoxia-induced apoptosis in chondrocytes.

## Materials and methods

### Cell culture and treatments

Human chondrocyte cell line, C28/I2, was purchased from American Type Culture Collection (ATCC) and were cultivated in Dulbecco’s modified Eagle’s medium (DMEM) (Gibco) containing 10% FBS (Invitrogen), 100 units/ml penicillin and 100 μg/ml streptomycin (Sigma–Aldrich) under 5% CO_2_ at 37°C. The cells were subcultured after reaching approximately 90% confluence. For hypoxia treatment, cells were placed in a hypoxia incubator with 5% CO_2_ and 3% oxygen. Oxygen concentration was monitored continuously (Forma 3130, Thermo Scientific). To overexpress RhoA in C28/I2 cells, the *Homo sapiens* RhoA coding sequence was cloned into the pcDNA3.1(+) vector (Invitrogen). And the RhoA-pcDNA3.1(+) or control pcDNA3.1(+) plasmid was transfected into C28/I2 cells with Lipofectamine 2000 (Invitrogen). Lysophosphatidic acid (LPA) (Santa Cruz Biotechnology) was utilized as an RhoA activator.

### MTT assay for cellular viability

Cellular viability of C28/I2 cells was examined by the MTT assay with MTT assay kit (Invitrogen). In brief, C28/I2 cells, post treatment, were added with MTT reagent for incubation at 37°C for 4 h, and then were updated with DMSO to dissolve the formazan crystals. Optical densities (OD) were measured at 450 nm by spectrophotometer (Bio–Rad). Cellular viability was presented as average OD_450_ value.

### Apoptosis assay and CASP 3 assay

Apoptosis induction in C28/I2 cells was assayed with the annexin V/FITC apoptosis detection kit (Abcam). In brief, 1 × 10^6^ C28/I2cells post treatment were stained with annexin V-FITC and propidium iodide and detected by an FACScan flow cytometer (BD Biosciences). The apoptosis was evaluated by a percentage of annexin V-positive cells to total cells.The CASP 3 activity in C28/I2 cells was examined with a Caspase SenSolyte kit (for CASP 3) (AnaSpec). Cells were collected and were washed for three times with ice-cold PBS, then were resuspended in 100 μl (final volume) of a caspase buffer solution supplemented with the fluorogenic peptide substrate Ac-DEVD-AMC for incubation for 30 min at 37°C. And the CASP 3 activity was determined by assessment of Asp-Glu-Val-Asp (DEVD)-AMC cleavage in a fluorescence spectrophotometer (Hitachi) with an excitation wavelength of 390 nm and an emission wavelength of 460 nm. And the activity was expressed as percent value of fluorescence intensity of AMC to control.

### Western blotting assay

The mitochondrial and cytosolic proteins in C28/I2 cells were isolated with the Mitochondria/Cytosol Fractionation Kit (Abcam) and were supplemented with a protease inhibitor cocktail (Roche). Then the protein samples were separated with SDS/PAGE (12% gel) and were electrotransferred on to nitrocellulose membranes (Millipore). The membranes were incubated by rabbit antibody against Cyt *c*, CASP 3, CREB, CREB with Ser^133^ phosphorylation, RhoA (all Santa Cruz Biotechnology) or β-actin (Sigma–Aldrich). Then, the specific binding signal was acquired using the ECL detection systems with the incubation with horseradish-peroxidase–conjugated secondary antibodies (Pierce).

### Measurement of mitochondrial superoxide and ROS

The mitochondrial superoxide was examined with MitoSOX™ Red mitochondrial superoxide indicator (Invitrogen) (Ex/Em =580/510 nm), which was highly selective and detects the superoxide in the mitochondria. C28/I2 cells were incubated with 5 μM MitoSOX™ Red at 37°C for 30 min and then were detected after the removal of the reagent and three times washing. Intracellular ROS level of was quantified by the ROS-sensitive fluorophore 5-(and-6)-chloromethyl-2, 7-dichlorodi-hydrofluorescein diacetate (Invitrogen) according to the product’s manual. Briefly, confluent C28/I2 cells were incubated with the 5-chloromethyl-2, 7-dichlorodihydrofluorescein diacetate probe at 37°C for 30 min and washed with PBS for three times. Then the cells were rinsed with HBSS and were measured using the fluorescence spectrophotometer (Hitachi) with an excitation wavelength of 488 nm and an emission wavelength of 530 nm. Results were presented as a percent level to control.

### Measurement of MMP and complex IV activity

TMRE–Mitochondrial Membrane Potential Assay Kit (Abcam) was used to determine the MMP in C28/I2 cells, and 3 × 10^5^ C28/I2 cells were incubated with the MMP-sensitive fluorescent TMRE for 30 min at 37°C (1000 nM FCCP was added to the positive control cells 10 min prior to TMRE). Cells were then trypsinized, centrifuged and resuspended in 0.4 ml of DPBS with 0.2% BSA and were analysed for TMRE fluorescence by the fluorescence spectrophotometer (Hitachi) with an excitation wavelength of 549 nm and an emission wavelength of 575 nm. Complex IV activity was measured by Complex IV Rodent Enzyme Activity Microplate Assay Kit (Abcam) according to manufacturer’s instructions. In brief, C28/I2 cells were collected and lysed with the detergent extraction. Then protein samples were serially diluted and were incubated at room temperature for 3 h in the plate in each well of which the enzyme-linked monoclonal antibody against complex IV has been immobilized. Then the binding was assayed by the fluorescence spectrophotometer (Hitachi) with an excitation wavelength of 549 nm and an emission wavelength of 575 nm. Results were presented as a percent level to control.

### Statistical analysis

Statistical analyses were performed using SPSS18.0 software (IBM SPSS). All data were expressed as mean ± S.E.M. Student’s *t* test was performed for the difference between two groups. A *P* value <0.05 was considered as statistically significant.

## Results

### Hypoxia induces apoptosis and inhibits CREB phosphorylation in chondrocytes

**Figure 1:**
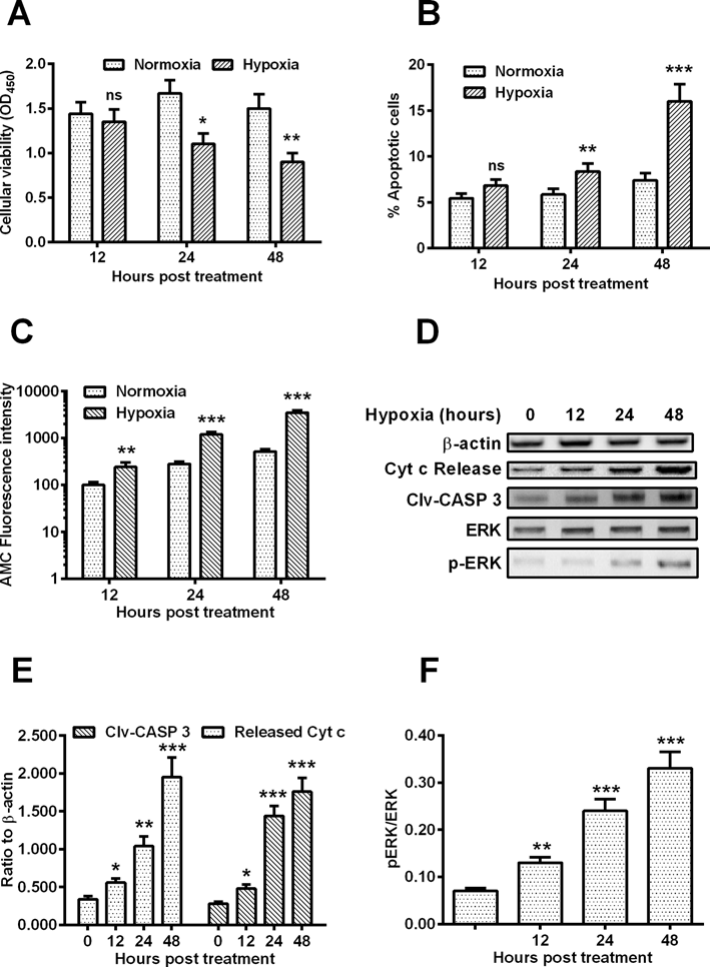
Cellular viability, apoptosis and CREB phosphorylation in the hypoxia-treated chondrocyte C28/I2 cells C28/I2 cells were inoculated under hypoxia or normoxia for 12, 24 or 48 h and were then analysed for the cellular viability with MTT assay, for the apoptosis with annexin V/FITC apoptosis detection kit, for the CASP 3 activity with AMC Caspase-3 Assay Kit and for the CREB phosphorylation with Western blotting assay. MTT assay results (**A**), Percentage of apoptotic cells (**B**), percentage of CASP 3 activity (**C**) of the C28/I2 cells under hypoxia or normoxia were presented respectively; (**D-F**) Western blotting assay of released Cyt *c* (**E**), cleaved CASP 3 (Clv-CASP 3) (**E**), CREB with p-CREB or without phosphorylation (CREB) (**F**) in the hypoxia-treated C28/I2 cells for 0, 12, 24 or 48 h. Each result was averaged for triple independent experiments. Statistical significance was shown as *P<0.05; **P<0.01 or ***P<0.001; ns, no significance.

In order to investigate the apoptosis induction by hypoxia in chondrocytes, we examined the cellular viability with MTT assay, the apoptosis induction with flow cytometric analysis and the CASP 3 activity with Caspase SenSolyte kit (for CASP 3) in the chondrocyte C28/I2 cells under hypoxia or normoxia. MTT assay results (Figure 1A) demonstrated that the hypoxia treatment for 24 or 48 h significantly reduced the viability of C28/I2 cells (*P*<0.05 or *P*<0.01). The apoptosis level was also significantly higher in the hypoxia-treated C28/I2 cells than in the C28/I2 cells under normoxia (Figure 1B, *P*<0.01 or *P*<0.001). The activity of active CASP 3, revealed by the fluorescence intensity AMC, in the hypoxia-treated C28/I2 cells was also significantly higher in the hypoxia group (Figure 1C) at 12, 24 or 48 h post treatment (H.P.T.) (*P*<0.01 or *P*<0.001).

Western blotting assay was also performed to examine the apoptosis-associated markers, such as released Cyt *c* and Clv-CASP 3 in the hypoxia- or normoxia-treated C28/I2 cells. Figure 1D indicated that there were significantly high levels of Cyt *c* release and Clv-CASP 3 (Figure 1E) in the hypoxia-treated C28/I2 cells at 12, 24 or 48 h post H.P.T. CREB has been well recognized to function as a prosurvival signal [18], and our study has also investigated the activation of CREB signalling in the hypoxia-treated C28/I2 cells (Figure 1F). The Western blotting assay also indicated that the phosphorylated CREB was also markedly down-regulated in the hypoxia-treated C28/I2 cells than in the normoxia-treated C28/I2 cells (Figure 1F), whereas the CREB without phosphorylation was not markedly regulated by hypoxia in C28/I2 cells. Taken together, we confirmed the apoptosis induction by hypoxia in chondrocyte C28/I2 cells with the down-regulation of CREB phosphorylation.

### Hypoxia induces mitochondrial dysfunction in chondrocyte C28/I2 cells

**Figure 2:**
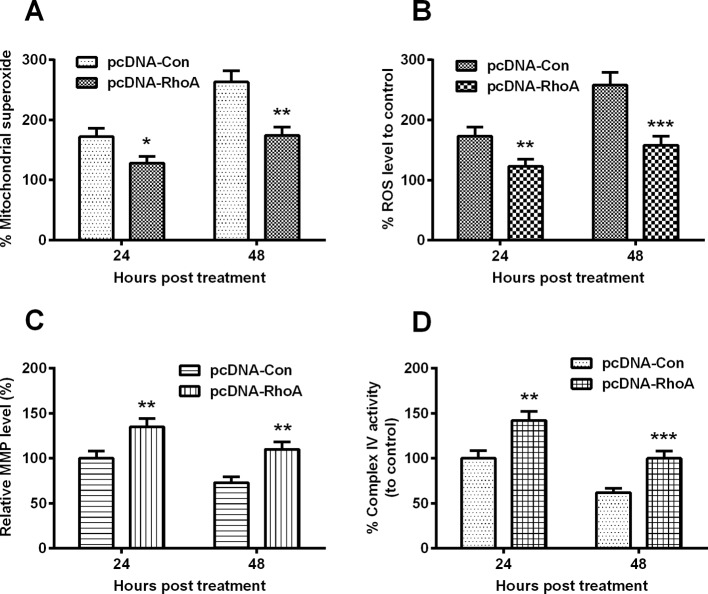
Mitochondrial dysfunction in the hypoxia-treated C28/I2 cells C28/I2 cells were inoculated under hypoxia or normoxia for 12, 24 or 48 h and then the percentage of mitochondrial superoxide levels (**A**), of ROS level (**B**), of MMP (**C**) and of Complex IV activity (**D**) were assayed respectively. All results were averaged for three independent experiments. *P<0.05; **P<0.01; ***P<0.001 or ns: no significance.

We then investigated whether hypoxia induced chondrocyte mitochondrial dysfunction. Firstly, we examined the superoxide generation, using MitoSOX, a live-cell-permeable and mitochondrial localizing superoxide indicator, in C28/I2 cells under hypoxia or normoxia. As shown in Figure 2A, there was a significantly high level of superoxide in the hypoxia-treated cells at both 24 and 48 H.P.T. (*P*<0.01 or *P*<0.001). Secondly, we measured ROS production in the hypoxia-treated C28/I2 cells. Figure 2B demonstrated that hypoxia enhanced the ROS production from 12 to 48 H.P.T. (*P*<0.05, *P*<0.01 or *P*<0.001). On the other side, MMP, as a well-confirmed indicator of mitochondrial function was significantly reduced in the hypoxia-treated C28/I2 cells at 24 or 48 H.P.T. (*P*<0.05 for 24 H.P.T. or *P*<0.01 for 48 H.P.T., Figure 2C). And the activity of mitochondrial respiratory chain complex IV was markedly down-regulated in the hypoxia-treated C28/I2 cells (Figure 2D), at 12, 24 or 48 H.P.T. (*P*<0.05, *P*<0.01 or *P*<0.001). Therefore, hypoxia promotes mitochondrial dysfunction in chondrocyte C28/I2 cells.

### RhoA overexpression reduces the hypoxia-induced CREB phosphorylation and apoptosis in C28/I2 cells

**Figure 3:**
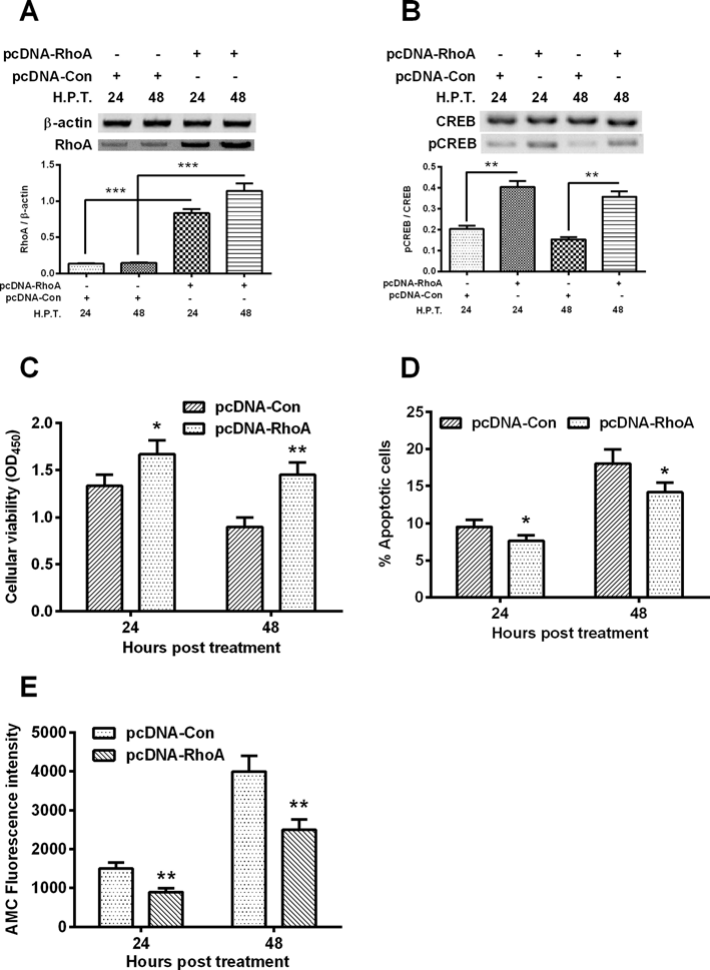
RhoA overexpression and its regulation on the hypoxia-induced apoptosis in C28/I2 cells C28/I2 cells were transfected with RhoA-pcDNA3.1(+) (pcDNA-RhoA) or with control pcDNA3.1(+) (pcDNA-Con) and were incubated for 24 or 48 h under hypoxia, then the RhoA in protein level (**A**), the CREB with or without phosphorylation (**B**), the cellular viability (**C**), percentage of apoptotic cells (**D**) and the CASP 3 activity (**E**) were assayed respectively. All experiments were performed independently in triplicate. Statistical significance was shown as *P<0.05; **P<0.01 or ***P<0.001; ns, no significance.

RhoA is a well-recognized apoptosis suppressor [18]. To further investigate the molecular pathway underlining the hypoxia-induced, CREB-associated apoptosis in chondrocytes, we then overexpress RhoA in the hypoxia-treated C28/I2 cells, and then re-evaluated the apoptosis induction and CREB phosphorylation. Figure 3A indicated that the RhoA was markedly promoted by the transfection with RhoA-pcDNA3.1(+) plasmid at both 24 and 48 h post transfection (*P*<0.001 respectively). Moreover, the p-CREB level was also significantly promoted by the RhoA-pcDNA3.1(+) transfection (Figure 3B, *P*<0.01 respectively for 24 or 48 H.P.T.). Then we investigated the regulation of the overexpressed RhoA on the hypoxia-induced apoptosis in C28/I2 cells. As shown in Figure 3C, the hypoxia-mediated viability reduction was markedly ameliorated by the RhoA-pcDNA3.1(+) transfection (*P*<0.05 for 24 H.P.T. or *P*<0.01 for 48 H.P.T.); and the RhoA overexpression also reduced the hypoxia-mediated apoptosis of C28/I2 cells (either *P*<0.05 for 24 or 48 h)(Figure 3D). In addition, CASP 3 activity, AMC fluorescence intensity in the hypoxia-treated C28/I2 cells was significantly inhibited in the RhoA overexpressrion group, than in the control group (*P*<0.05 for 24 h or *P*<0.01 for 48 h post transfection ,Figure 3E). Therefore, RhoA overexpression attenuated the hypoxia-induced apoptosis in chondrocyte C28/I2 cells.

### RhoA overexpression reduces the hypoxia-induced mitochondrial dysfunction in chondrocytes

**Figure 4:**
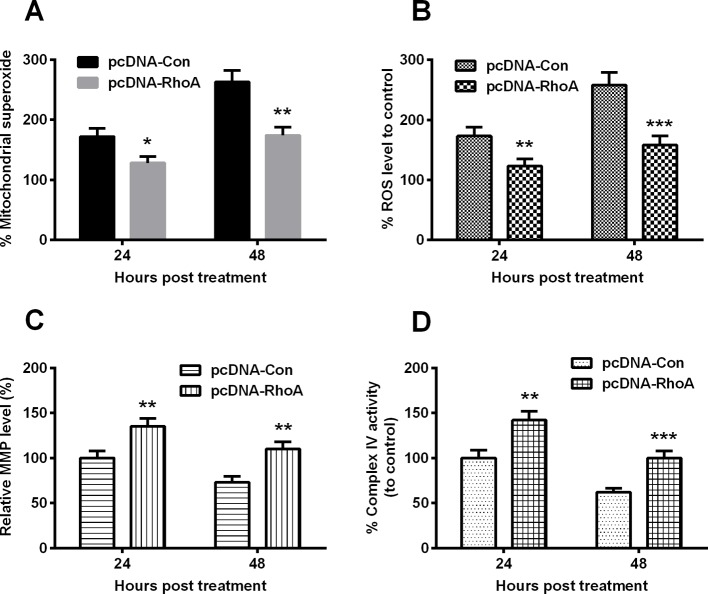
Mitochondrial dysfunction in the hypoxia-treated C28/I2 cells, post the RhoA overexpression C28/I2 cells were transfected with pcDNA-RhoA or with pcDNA-Con and were inoculated under hypoxia for 24 or 48 h and then the percentage of mitochondrial superoxide levels (**A**), of ROS level (**B**), of MMP (**C**) and of Complex IV activity (**D**) were assayed respectively. All results were averaged for three independent experiments. *P<0.05, **P<0.01 or ***P<0.001.

We then investigated the regulation of RhoA overexpression on the hypoxia-induced mitochondrial dysfunction. Firstly, we examined the mitochondrial superoxide level and the ROS generation in the hypoxia-treated C28/I2 cells, which were transfected with RhoA-pcDNA3.1(+) or control pcDNA3.1(+) plasmid. Figure 4A indicated that the promoted mitochondrial superoxide production was significantly reduced in the RhoA-pcDNA3.1(+)-transfected C28/I2 cells under hypoxia than in the control pcDNA3.1(+) plasmid-transfected C28/I2 cells under hypoxia (Figure 4A, *P*<0.05 for 24 or *P*<0.01 for 48 h post transfection). And the ROS release was also blocked by the RhoA overexpression (Figure 4B, *P*<0.01 for 24 or *P*<0.001 for 48 h post transfection). Moreover, the hypoxia-mediated inhibition of MMP and mitochondrial respiratory chain complex IV was markedly ameliorated by the RhoA overexpression (Figure 4C,D, *P*<0.01 or *P*<0.001). Taken together, our results showed a mitochondrial protection of RhoA overexpression in the hypoxia-treated C28/I2 cells.

### RhoA activator LPA reduces the hypoxia-induced apoptosis in C28/I2 cells via ameliorating mitochondrial dysfunction

**Figure 5:**
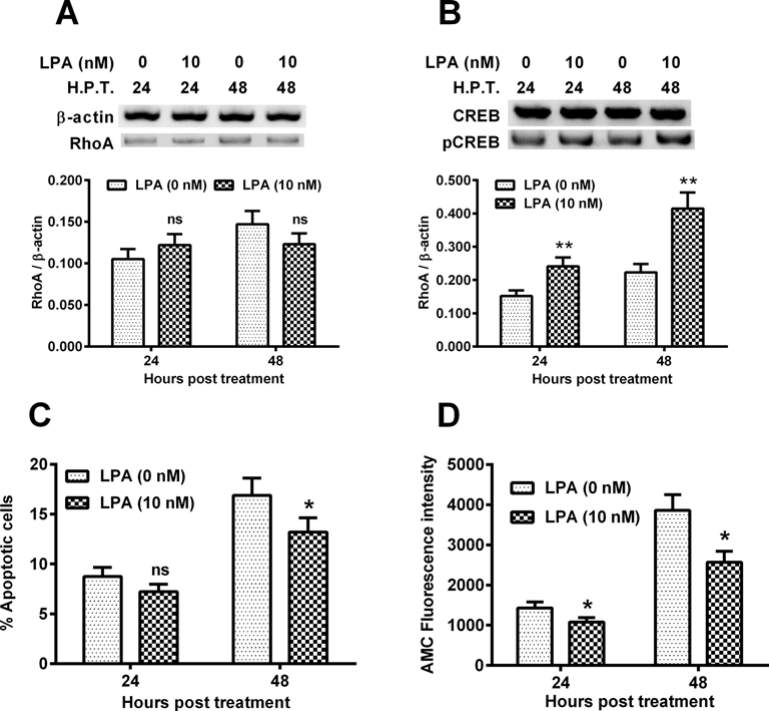
LPA reduces the hypoxia-induced apoptosis in C28/I2 cells C28/I2 cells were treated with 0 or 10 nM LPA and then were incubated for 24 or 48 h under hypoxia, then the RhoA in protein level (**A**), the CREB with or without phosphorylation (**B**), the percentage of apoptotic cells (**C**) and the CASP 3 activity (**D**) were assayed respectively. All experiments were performed independently in triplicate. Statistical significance was shown as *P<0.05 or **P<0.01; ns: no significance.

To reconfirm the inhibitory role of RhoA on the hypoxia-induced mitochondrial dysfunction and apoptosis in C28/I2 cells, we then pretreated C28/I2 cells with 10 nM LPA and then re-evaluated the hypoxia-induced apoptosis and mitochondrial dysfunction. It was shown in Figure 5A that the LPA treatment did not regulate the RhoA expression. However, the RhoA-induced CREB phosphorylation was significantly promoted by the LPA treatment (*P*<0.01 respectively at either 24 or 48 H.P.T., Figure 5B). Moreover, the hypoxia-mediated apoptosis was also markedly reduced in the LPA-treated C28/I2 cells at 48 H.P.T. (10 nM) (*P*<0.05, Figure 5C). AMC fluorescence intensity in the hypoxia-treated C28/I2 cells was significantly inhibited by the 10 nM LPA at 24 or 48 H.P.T. (*P*<0.05 respectively, Figure 5D). Therefore, RhoA activation by LPA also attenuated the hypoxia-induced apoptosis in chondrocyte C28/I2 cells.

**Figure 6:**
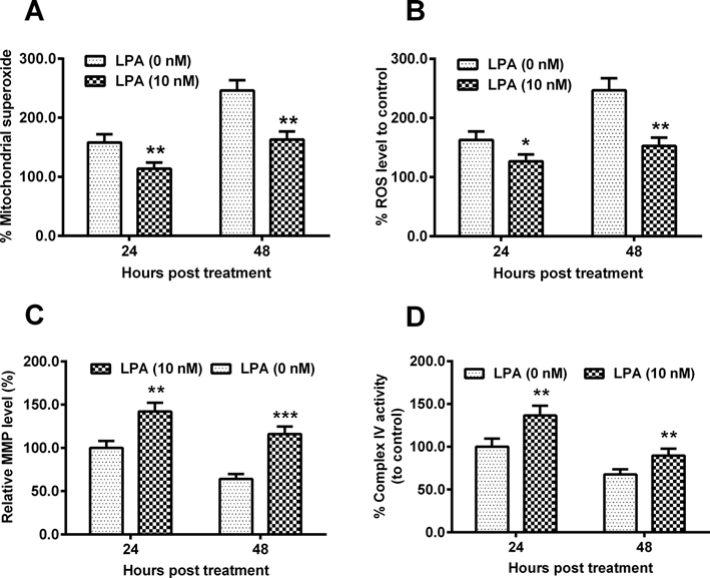
LPA ameliorates the hypoxia-induced mitochondrial dysfunction in C28/I2 cells C28/I2 cells were treated with 0 or 10 nM LPA and then were incubated for 24 or 48 h under hypoxia, and then the percentage of mitochondrial superoxide levels (**A**), of ROS level (**B**), of MMP (**C**) and of Complex IV activity (**D**) were assayed respectively. All results were averaged for three independent experiments. *P<0.05, **P<0.01 or ***P< 0.001.

In addition, we also examined the regulation by LPA on the hypoxia-induced mitochondrial dysfunction. Results demonstrated that both the hypoxia-induced superoxide production (Figure 6A) and the ROS release (Figure 6B) were markedly reduced in the LPA-treated (10 nM) C28/I2 cells than in the control C28/I2 cells (*P*<0.05 or *P*<0.01). On the other side, the hypoxia-mediated inhibition of both MMP and mitochondrial respiratory chain complex IV was significantly ameliorated by the LPA treatment (*P*<0.01 or *P*<0.001, Figure 6C,D). Thus, our results reconfirmed the involvement of mitochondrial dysfunction in the RhoA-mediated inhibition of hypoxia-induced apoptosis in C28/I2 cells.

## Discussion

Oxygen affects the activity of multiple types of cells and is involved in many processes that are important for degenerative bone disease [21–23]. The current studies found that hypoxia promoted apoptosis in the chondrocyte C28/I2 cells, via up-regulating CASP 3 activity and via up-regulating inducing mitochondrial dysfunction. Apoptosis can be initiated through membrane receptor-associated and mitochondrial-initiated pathways that converge and mediate their downstream apoptosis effects [24]. Therefore, the mitochondria dependence was confirmed in the hypoxia-induced apoptosis in C28/I2 cells. Mitochondrial dysfunction such as the overeproduction of mitochondrial superoxide and ROS has been identified to be one of the important mechanisms in the hypoxia-mediated damage to chondrocytes [23,25,26]. On the other side, hypoxia inhibits complex I, III or IV [27] and MMP [28] in chondrocytes. Our study confirmed the high promotion of mitochondrial superoxide and ROS and the inhibition of MMP and complex IV in the hypoxia-treated C28/I2 cells.

Oxidative stress has been implicated in the pathogenesis of osteoporosis [29]. It is conceivable that mitochondrial-mediated ROS generation under oxidative stress leads to damage to ROS-produced cells or peripheral chondrocytes [30]. The present study demonstrated that the hypoxia also significantly exerted mitochondrial dysfunction in chondrocyteic C28/I2 cells. Hypoxia has been implicated in the pathogenesis of osteoporosis [31,32], at least partly via inducing the mitochondrial dysfunction [30,33]. Mitochondrial-mediated ROS generation under oxidative stress leads to damage of ROS produced cells or peripheral chondrocytes [30]. The Allicin treatment ameliorated the H_2_O_2_-mediated repression of CREB signalling [30]. Previously, PI3K and CREB were shown to be implicated in chondrocyte-like cell proliferation and differentiation [34,35]. In the present study, RhoA overexpression in C28/I2 cells also promoted the CREB signalling and thus ameliorated the hypoxia-induced mitochondrial dysfunction and apoptosis.

Multiple bone anabolic agents have been confirmed to prevent chondrocyte apoptosis, such as parathyroid hormone (PTH) [36] and insulin-like growth factor-I [37]. RhoA has been well recognized to promote cell survival and survival-related signalling [38,39], such as increased Bcl-2 expression [17] via binding and hydrolysing GTP. The signalling pathway through which RhoA prevents apoptosis varies in different tissues. And our results confirmed that the hypoxia-induced apoptosis was inhibited by the RhoA overexpression, implying that RhoA signalling is implicated in the antiapoptosis response in chondrocytes. RhoA is one number of Rho small GTPase families [40]. Activated Rho GTPase induces a variety of cellular responses [41,42]. RhoA has been suggested to modulate the cell cycle and survival [43,44] of cancer cells. Moreover, RhoA has implicated several antiapoptotic pathways in the suppression of apoptosis [45] via activating ERK [45], via up-regulating antiapoptotic Bcl-2 expression [46,47] or via regulating p53 pro-apoptotic pathway [48]. More recently, RhoA has been identified to regulate the CREB activity [49]. And in the present study, we found that RhoA also exerted a protective role against the hypoxia-induced apoptosis in chondrocytes via regulating the CREB phosphorylation.

In conclusion, the present study confirmed the reduced CREB phosphorylation, along with the apoptosis induction and mitochondrial dysfunction in the hypoxia-treated chondrocyte cells. And the overexpression of RhoA ameliorated the hypoxia-induced mitochondrial dysfunction and apoptosis via blocking the reduction in CREB phosphorylation.

## Abbreviations

AMC: Aluminum matrix composite
Bcl-2: B-cell
CASP 3: caspase 3
Clv-CASP 3: cleaved CASP 3
CREB: cAMP response element-binding protein
Cyt *c*: cytochrome c
DPBS: Dulbecco's phosphate Buffered Saline
ERK: Extracellular signal regulated kinases
FCCP: Mitochondrial oxidative phosphonylation inhibitor
HBSS: Hank's balanced salt solution
H.P.T.: hours post treatment
LPA: lysophosphatidic acid
MMP: mitochondrial membrane potential
OD: Optical density
PI3K: PHOSPHATIDYLINOSITOL3-KINASE
ROS: reactive oxygen species
TMRE: Tetramethylrhodamine ethylester
